# Population-specific optimization of polygenic risk scores for breast cancer in East Asians

**DOI:** 10.1016/j.gimo.2026.104487

**Published:** 2026-08-03

**Authors:** Shu-Hsuan Liu, Yu-Chun Wang, Yun-Jer Shieh, Hai-Feng Tan, Kuo-Jang Kao, Chih-Chiang Hung, Ming-Feng Hou, Chi-Cheng Huang, Ching-Hung Lin, An-Chieh Feng, Jeng-Fong Chiou, Chien-Hsiun Chen, Ling-Hui Li, Taipei Medical University Hospital Consortium, Hsing-Lung Chao, Tsu-Yi Chao, Hsin-An Chen, Shyr-Ming Sheen Chen, Yin-Ju Chen, Jeng-Fong Chiou, Tzeon-Jye Chiou, Chia-Lun Chang, Wei-Wen Chang, Wei-Kai Chuang, Po-Wei Huang, Chun-Yu Hsieh, Chin-Sheng Hung, Jia-Jun Lai, Kuan-Der Lee, Hsin-Lun Lee, Ming-Hsien Li, Minhua Liao, Huan-Tze Lin, Jang-Chun Lin, Yung-Feng Lin, Cheng-Sheng Liu, Long-Sheng Lu, Yu-Hao Sheng, Chih-Ming Su, Jo-Ting Tsai, Jia-Ruey Tsai, Lai-Lei Ting, Shih-Hsin Tu, Huey-En Tzeng, Hsin-Hsien Yu, Sang-Hue Yen, Wen-Ke Wang, Jeng-You Wu, Chia-chun Kuo, Tzu-Hung Hsiao, Yu-Hsuan Joni Shao

**Affiliations:** 1 Department of Medical Research, Taichung Veterans General Hospital, Taichung, Taiwan; 2 Graduate Institute of Biomedical Informatics, College of Medical Science and Technology, Taipei Medical University, Taipei, Taiwan; 3 Institute of Public Health, National Yang-Ming Chiao-Tung University, Taipei, Taiwan; 4 Institute of Statistical Science, Academia Sinica, Taipei, Taiwan; 5 Departments of Research and Molecular Medicine, Koo Foundation Sun Yat-Sen Cancer Center, Taipei, Taiwan; 6 Division of Breast Surgery, Department of Surgery, Taichung Veterans General Hospital, Taichung, Taiwan; 7 Division of Breast Oncology and Surgery, Department of Surgery, Kaohsiung Medical University Hospital, Kaohsiung Medical University, Kaohsiung, Taiwan; 8 Comprehensive Breast Health Center, Taipei Veterans General Hospital, Taipei, Taiwan; 9 Division of Breast Surgery, Department of Surgery, Taipei Veterans General Hospital, Taipei, Taiwan; 10 Institute of Epidemiology and Preventive Medicine, College of Public Medicine, National Taiwan University, Taipei, Taiwan; 11 Department of Oncology, National Taiwan University Hospital, Taipei, Taiwan; 12 Department of Medical Oncology, National Taiwan University Cancer Center Hospital, Taipei, Taiwan; 13 Division of General Surgery, Department of Surgery, Tri-Services General Hospital, National Defense Medical Center, Taipei, Taiwan; 14 Department of Radiation Oncology, Taipei Medical University Hospital, Taipei, Taiwan; 15 Institute of Biomedical Sciences, Academia Sinica, Taipei, Taiwan; 16 Department of Public Health, Fu Jen Catholic University, New Taipei City, Taiwan; 17 Institute of Genomics and Bioinformatics, National Chung Hsing University, Taichung, Taiwan; 18 Clinical Big Data Research Center, Taipei Medical University Hospital, Taipei, Taiwan; 19 Health and Clinical Research Data Center, Office of Data Science, Taipei Medical University, Taipei, Taiwan

**Keywords:** Asian ancestry, Breast cancer, Polygenic risk score, Population transferability

## Abstract

**Purpose:**

Polygenic risk scores (PRS) have shown promise in predicting disease susceptibility, but their accuracy varies across populations due to the limited representation of non-European ancestries in genomic studies. While European-derived PRS demonstrate some transferability to Asian populations, optimizing these scores for specific populations remains crucial for improving risk prediction.

**Methods:**

Using data from the Taiwan Precision Medicine Initiative (*n* = 46,422) and an external validation cohort (*n* = 891), we developed an approach to optimize European-derived PRS for breast cancer prediction in East Asians. Our method involved 2 key steps: (1) replacing European PRS variants with Asian-specific single nucleotide polymorphisms within the same linkage disequilibrium blocks (*r*^2^ > 0.8) and (2) incorporating additional population-specific variants. We validated this approach using both breast and prostate cancer data sets.

**Results:**

The optimized PRS (PRS_EUR_replace+TPMI_) showed improved predictive performance compared with the original European PRS (PRS_EUR_) in our main cohort, with an increased area under the curve from 0.608 to 0.617 and odds ratios per SD from 1.47 (95% CI 1.36-1.60) to 1.51 (95% CI 1.39-1.63). Individuals in the top 5% of PRS_EUR_replace+TPMI_ demonstrated a 2.09-fold increased risk of developing breast cancer compared with those in the 40^th^ to 60^th^ percentile. External validation confirmed enhanced performance, and similar improvements were observed when this method was applied to prostate cancer prediction.

**Conclusion:**

Our study presents a practical approach for optimizing European-derived PRS in non-European populations, which is particularly beneficial for populations with limited genetic data. As genetic data collection expands in these populations, this approach may help reduce health care disparities in the implementation of precision medicine.

## Introduction

Polygenic risk scores (PRS) are a quantitative measure used to assess an individual’s genetic susceptibility to certain diseases by aggregating the cumulative effects of multiple genetic variants across the genome. They may potentially serve as a critical instrument in advancing precision medicine by providing a comprehensive assessment of an individual’s genetic susceptibility to complex traits and diseases. However, the majority of PRS models are built upon data predominantly collected from individuals of European descent. The PRS derived from these populations may have limited predictability for other ethnic populations due to differences in allele frequencies and linkage disequilibrium (LD).^1^^,^^2^ Despite the fact that several Asian biobanks, such as Japanese, Korean, and Han Chinese, have been established, substantial genomics data are lacking in the Asian population, which poses a challenge in understanding the transferability and clinical utility of PRS models in underrepresented populations.

Recent studies showed that PRSs for coronary artery disease, type 2 diabetes, and breast and prostate cancer had similar accuracy across European and Asian populations.^3^^,^^4^ Using the same PRS developed in European populations, the estimated odds ratio (OR) per unit increase of standardized PRSs for overall breast cancer was 1.52 (95% CI 1.49-1.56) in an Asian cohort, compared with 1.61 (95% CI 1.57-1.66) in a cohort from European studies.^3^ The findings suggest that the PRS developed from European ancestry data can serve as a valuable foundation for predicting cancer risk in Asian populations. The predictability of these PRSs gives hope to diminish the healthcare disparity due to the underrepresentation of genetic data from the Asian population. However, given the ethnic diversity in Asia, population-specific PRS is still limited, and it is pivotal in identifying high-risk populations to accelerate risk stratification. Several studies have emerged to address population-specific calibration in risk prediction. One innovative approach combines multiple data sets to not only expand sample size but also leverage Polygenic Risk Score–Continuous Shrinkage, cross-population, a method that integrates genome-wide association study (GWAS) summary statistics from multiple populations for PRS development.^5^ Another study investigated PRS optimization by incorporating multiple strategies, while simultaneously integrating nongenetic risk factors to enhance predictive accuracy.^6^ However, these studies face practical challenges in PRS optimization, primarily stemming from constrained sample sizes and the computational complexity of existing methodologies. The limited specificity of current approaches for distinct Asian populations highlights the critical need for more targeted and nuanced solutions in genetic risk prediction.

The incidence of breast cancer, as well as other cancers, is increasing dramatically among Asians, who make up more than half of the world’s population.^7^ The estimated incidence of new cancer cases in Asia is projected to increase from 6.1 million in 2008 to 10.6 million in 2030, driven by aging populations and lifestyle changes linked to economic development in the region.^8^ Large-scale genetic data sets from local populations are growing, necessitating innovative approaches to improve the accuracy of PRSs across diverse populations. Current limitations in population-specific genetic data highlight the need for robust methodologies that bridge knowledge gaps in genetic risk assessments.

In this study, we demonstrated an approach to optimize one of the best-performing European-derived PRS for breast cancer prediction in an East Asian population. We evaluated the clinical utility and performance of the optimized PRS, leading to more robust and reliable disease risk predictions. To validate the generalizability of our methodology, we further applied this approach to a prostate cancer data set, confirming its effectiveness in risk prediction for Asian populations. Our findings not only enhance the cross-population applicability of existing PRS but also demonstrate the potential for implementing population-adjusted genetic risk assessment in personalized health care. This study provides a systematic framework for adapting European-derived PRS to non-European populations, addressing a critical gap in genomic medicine and moving toward more equitable precision health care.

## Materials and Methods

### Study population

We used the Taiwan Precision Medicine Initiative (TPMI) data to derive and validate our PRS and another data set collected from 2 hospitals in Taiwan for external validation. TPMI is held by Academia Sinica in partnership with 16 top medical centers in Taiwan and has established a database consisting of comprehensive clinical data and genetic profiles of around half a million participants.^9^ A total of 7737 breast cancer cases and 38,685 age- and sex-matched controls from the TPMI data set were included in this study. We divided the data into set 1, which comprised 6887 cases and 34,435 controls for developing our own PRS and effect sizes of single-nucleotide polymorphisms (SNPs), and set 2, which comprised 850 cases and 4250 controls for establishing our optimization method. In addition, we used an external data set with 151 cases and 740 controls, collected from 2 hospitals, for validating our approach. The descriptive statistics for the 2 populations are shown in Table 1. All research investigations received approval from the appropriate institutional ethics committees and review boards. All participants provided written informed consent prior to their involvement in the studies.

**Table 1:** Basic characteristics of study population

Characteristic	All Samples After QC Step	Set 1 (A, B)	Set 2 (C, D)	External Data Set
Sample size, *n*	46,422	41,322	5100	891
Case, *n*	7737	6,887	850	151
Control, *n*	38,685	34,435	4250	740
Age at onset, mean (SD)	54.68 (10.26)	54.74 (10.29)	54.23 (10.11)	34.79 (3.92)

*QC*, quality control.

### Genotyping methods

All samples in this study were genotyped using the genotyping array designed by the Taiwan Biobank research group, TWB 2.0, a high-throughput Affymetrix Axiom genotyping platform that is specifically designed for the Taiwanese population.^10^ A total of 717,510 probes were provided on a single array of 686,463 SNPs covering all human chromosomes. All of them were selected because they were either clinically significant or immune-related, disease-related, or cancer-associated risk variants in the previous study.

The processing methods of the experiment were all implemented following relevant guidelines and regulations provided by Affymetrix, Inc. DNA extraction and purification were carried out through the QIAsymphony automatic nucleic acid extraction system (QIAGEN) with special magnetic bead technology. Genetic variants were imputed using the East Asian reference panel from the 1000 Genomes Project.^11^^,^^12^ Any SNPs that had an imputation quality score below 0.9 were excluded from the analysis.^12^ Quality control measures for both samples and genetic markers were conducted following the protocol published by Yang et al.^13^ During the quality control process, samples showing genetic relatedness were identified and removed based on identity by descent calculations. Figure 1 provides the workflow of the methods and data sets. The approaches for SNP selection to be included in PRS are described below. The lists of SNPs and the weights for the PRS computation are given in Supplemental Table 1.

**Figure 1:**
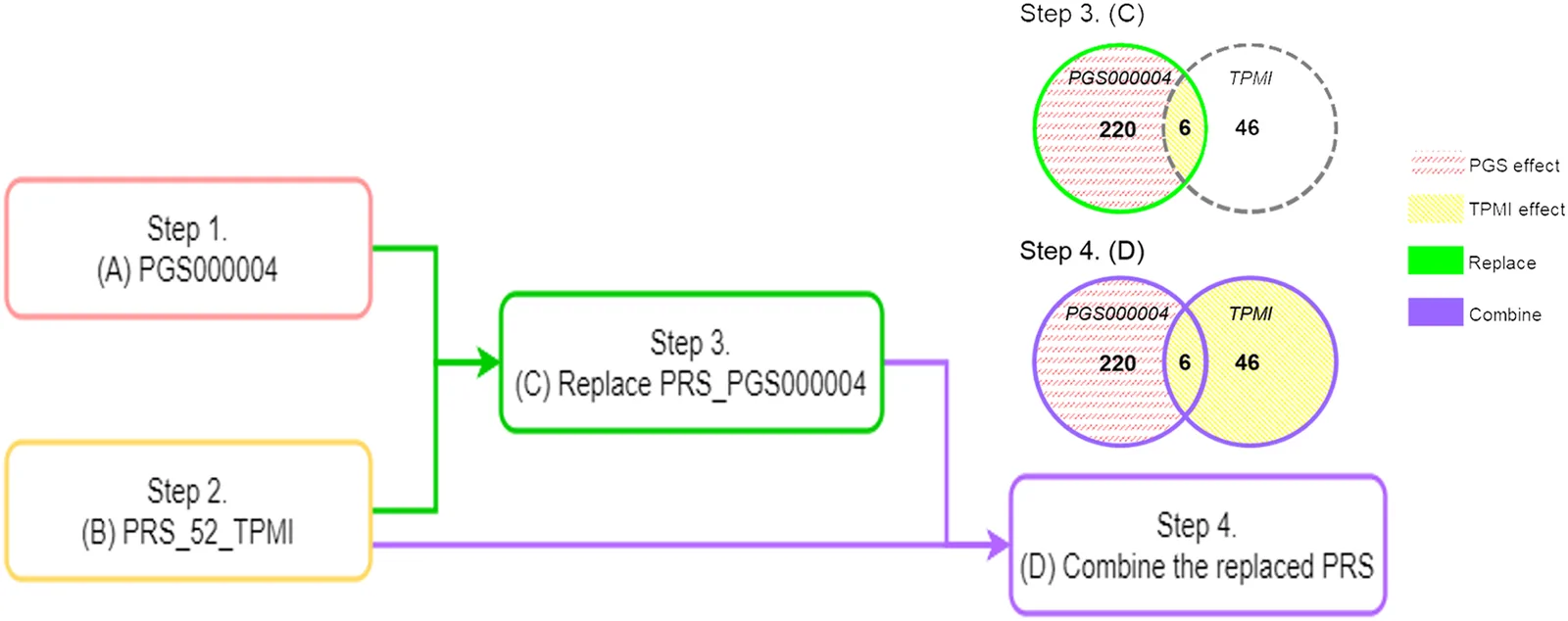
**Workflow for PRS development and SNP substitution strategy, with Venn diagram visualization of SNP distribution patterns across sample cohorts.** PGS, polygenic score; PRS, polygenic risk score; SNP, single-nucleotide polymorphism; TPMI, Taiwan Precision Medicine Initiative.

### GWAS and PRS_TPMI_ development

Using data set 1, a genome-wide association analysis was conducted using the TPMI data to evaluate the risk of common variants associated with breast cancer and the polygenic effect using PRS. We used an age-matched case-control approach and processed the data. Multiple quality control steps and genotype imputation were performed as mentioned above. Genetic association testing was done by PLINK^14^/PLINK2^15^ and a list of statistically significant (*P* < 1×10^−5^) SNPs associated with breast cancer across the genomes was generated. SNP clumping was conducted to remove highly correlated SNPs (pairwise correlation *r*^2^ > 0.5). PRS_TPMI_ was generated from C+T and was estimated using the following method.

$$Polygenic\;Risk\;Score=\beta_{1}X_{1}+\beta_{2}X_{2}+{\ldots \ldots .+\beta }_{m}X_{m}$$

β is the effect size of each SNP; X is the genotype encoding of an individual at the SNP.

Data set 1 was also used to calculate a PRS_313_ derived from the 313-SNP PRS (PGS000004) developed in women of European ancestry^16^ which is one of the best-performing risk predictions for the risk of breast cancer currently. Of the 313 SNPs developed for the European population, only 226 SNPs (PRS_226_EUR_) with an imputation quality score > 0.9 in our data set 1 remained in the analysis.

### PRS optimization strategy

1) LD-based replacement of European PRS variants with Asian-specific SNPs.

Few SNPs in PRS_EUR_ are in the same LD block as those in PRS_TPMI_. We replaced SNPs in PRS_EUR_ that were highly *r*^2^ correlated with those in PRS_TPMI_. High *r*^2^ indicates that 1 SNP may be in linkage with the other. In data set 2, we examined the performance of PRS_EUR_replace_ using *r*^*2*^ cutoffs of > 0.8, 0.5, and 0.3.

We used the LDmatrix Tool from the NIH’s online LDLink platform (https://ldlink.nih.gov/?tab=home) to generate LD matrices for the exchanged loci in our study. The process involved identifying the precise physical positions of the variants and retrieving all SNPs located between these 2 points. Population selection was performed directly within the online tool by choosing the corresponding ancestry group. Correlation analysis between TPMI-derived SNPs and those from PGS000004 was performed using the PLINK --ld-snp-list command.

2) Integrating population-specific variants into European PRS with substituted SNPs.

To enhance the predictive performance of the PRS, we further combined PRS_EUR_replace_ with the rest of the SNPs discovered in PRS_TPMI_. PRS_EUR_replace_ was supplemented with TPMI-exclusive variants, allowing the combined score to incorporate a broader set of genetic signals. PRS_EUR_replace+TPMI_ generated using this approach was also tested in data set 2. In approaches 1 and 2, we considered combination weights for PRS derived from either PRS_EUR_ or PRS_TPMI_ without reweighting. Several approaches were tested, but we presented the method that gave the best area under the curve (AUC) in the study. The predictive performance of PRSs developed from these 2 approaches was assessed using AUC and OR per SD. We also estimated the OR for the population with the highest 5% and 10% PRS to examine the predictive ability of these PRSs.

### External validation

The performance of PRS_EUR_, PRS_TPMI_, PRS_EUR_replace_, and PRS_EUR_replace+TPMI_ was evaluated in an external data set. AUC, OR per SD, and the OR for the population with the highest 5% and the 10% PRS of the above 4 PRSs were estimated.

### Replication of the method in prostate cancer

To test the robustness of our method, we adopted the same approach in a prostate cancer cohort. We evaluated this approach in a cohort comprising 3478 prostate cancer cases and 17,390 controls (total *n* = 20,868), detailed in Supplemental Table 2. We generated a PRS for prostate cancer by combining SNPs from PGS003415,^17^ a polygenic score (PGS) comprising 268 genome-wide variants originally developed using a discovery cohort of more than 20,000 European-ancestry males, with 197 SNPs identified from our own study population. In addition, a population-specific PRS was constructed independently from our cohort to further evaluate the performance of our proposed approach.

## Results

The descriptive statistics for the data set included in our study are shown in Table 1. These include 2 independent populations, 1 with 46,422 individuals in our main population and the other with 891 individuals in our external population. Figure 1 describes our approach and the data set that we used in each procedure. Table 2 shows the results from different PRSs. The PRSs were rescaled and have a mean of 0 and an SD of 1. In PRS_EUR_, we adopted PGS000004 and obtained the weights directly from the PGS catalog. Only 226 SNPs with an imputation quality score > 0.9 were retained in the analysis. Table 2 shows that the ORs per SD in PRS_EUR_ is 1.47 (95% CI1.36-1.60; AUC = 0.608). In PRS_TPMI_ using the C+T approach in our population, the best PRS was obtained at a *P* value threshold of 1 × 10^−5^ and *r*^2^ = 0.5, resulting in a 52-SNP PRS (PRS_TPMI_) with OR_perSD_ (95% CI) of 1.37 (1.26-1.48; AUC = 0.587). Compared with patients who have PRS_TPMI_ between 40% and 60%, patients in the top 5% and 10% of PRS_TPMI_ demonstrated a 1.66-fold and 1.52-fold risk of developing breast cancer, respectively (Table 2).

**Table 2:** A demonstration of PRS approaches for breast cancer risk assessment in Taiwanese women

PRS Model	Method	SNP #	Effect Size	OR_perSD_ (95% CI)	AUROC	OR_Top 5_ (95% CI)	OR_Top 10_ (95% CI)
(A) PRS_EUR_	PGS000004	226	PGS catalog	1.47 (1.36-1.60)	0.608	2.15 (1.51-3.04)	1.87 (1.40-2.48)
(B) PRS_TPMI_	Clumping and thresholding R^2^>0.5, p-value<1e-5	52	TPMI	1.37 (1.26-1.48)	0.587	1.66 (1.16-2.36)	1.52 (1.14-2.01)
(C-1) PRS_EUR_replace_	Replace 6 SNPSs with LD threshold r^2^ ≥ 0.8 in (B)	225	PGS catalog TPMI	1.48 (1.36-1.60)	0.609	2.31 (1.64-3.23)	1.86 (1.40-2.45)
(C-2) PRS_EUR_replace10_	Replace 10 SNPSs with LD threshold r^2^ ≥ 0.5 in (B)	225	PGS catalog TPMI	1.49 (1.38-1.62)	0.611	2.10 (1.48-2.97)	1.73 (1.30-2.30)
(C-3) PRS_EUR_replace17_	Replace 17 SNPSs with LD threshold r^2^ ≥ 0.3 in (B)	231	PGS catalog TPMI	1.47 (1.36-1.60)	0.607	2.33 (1.66-3.25)	1.92 (1.46-2.53)
(D-1) PRS_EUR_replace+TPMI_	Combine (C-1) + (B)	269	PGS catalog TPM	1.51 (1.39-1.63)	0.617	2.09 (1.47-2.96)	1.93 (1.45-2.55)
(D-2) PRS_EUR_replace10+TPMI_	Combine (C-2) + (B)	266	PGS catalog TPM	1.51 (1.40-1.64)	0.618	2.29 (1.61-3.24)	1.98 (1.49-2.62)
(D-3) PRS_EUR_replace17+TPMI_	Combine (C-3) + (B)	265	PGS catalog TPM	1.52 (1.40-1.64)	0.619	2.49 (1.75-3.52)	2.08 (1.56-2.76)

*AUROC*, area under the receiver operating characteristic curve; *LD*, linkage disequilibrium; *OR*, odds ratio; *ORperSD*, odds ratio per SD; *ORTop 5*, odds ratio for the top 5%; *ORTop 10*, odds ratio for the top 10%; *PGS*, Polygenic Score; *PRS*, polygenic risk score; *SNP*, single-nucleotide polymorphism; *TPMI*, Taiwan Precision Medicine Initiative.

### PRS_EUR_ replacement of Asian SNPs by LD

In the process of developing PRS_TPMI_, we found that several SNPs in PRS_226_EUR_ and PRS_TPMI_ are in the same linkage LD block, where the allele of 1 polymorphism in an LD block (haplotype) can predict the allele of an adjacent polymorphism. We replaced 6 SNPs in PRS_EUR_ in the same LD block with those in PRS_TPMI_ and generated PRS_EUR_replace_. The LD threshold used for pruning was 0.8. The OR_perSD_ (95% CI) of PRS_EUR_replace_ was 1.48 (1.36-1.60; AUC = 0.609). The replacement strategy is illustrated in Supplemental Figure 1, and the performance of PRS using different LD thresholds is shown in Table 2 (C-1 to C-3). Compared with individuals who have PRS_EUR_replace_ between 40% and 60%, individuals in the top 5% and 10% of PRS_EUR_replace_ demonstrated a 2.31-fold and 1.86-fold risk of developing breast cancer, respectively (Table 2).

### Combination of PRS_EUR_replace_ and PRS_TPMI_

We combined the rest of the SNPs in PRS_TPMI_ with PRS_EUR_replace_, resulting in a PRS with 269 SNPs (PRS_EUR_replace+TPMI_). The OR_perSD_ (95% CI) of PRS_EUR_replace+TPMI_ was 1.51 (1.39-1.63; AUC = 0.617). The performance of PRS using different LD thresholds is shown in Table 2 (D-1 to D-3). In comparison to individuals with PRS_EUR_replace+TPMI_ falling within the 40% and 60% range, those positioned in the top 5% and 10% of PRS_EUR_replace+TPMI_ exhibited a 2.09-fold and 1.93-fold increased likelihood of developing breast cancer, respectively (Table 2). ORs of individuals in each PRS quantile in predicting the risk and area under the receiver-operating characteristic curve (AUROC) of developing breast cancer by different PRSs are presented in Figure 2.

**Figure 2:**
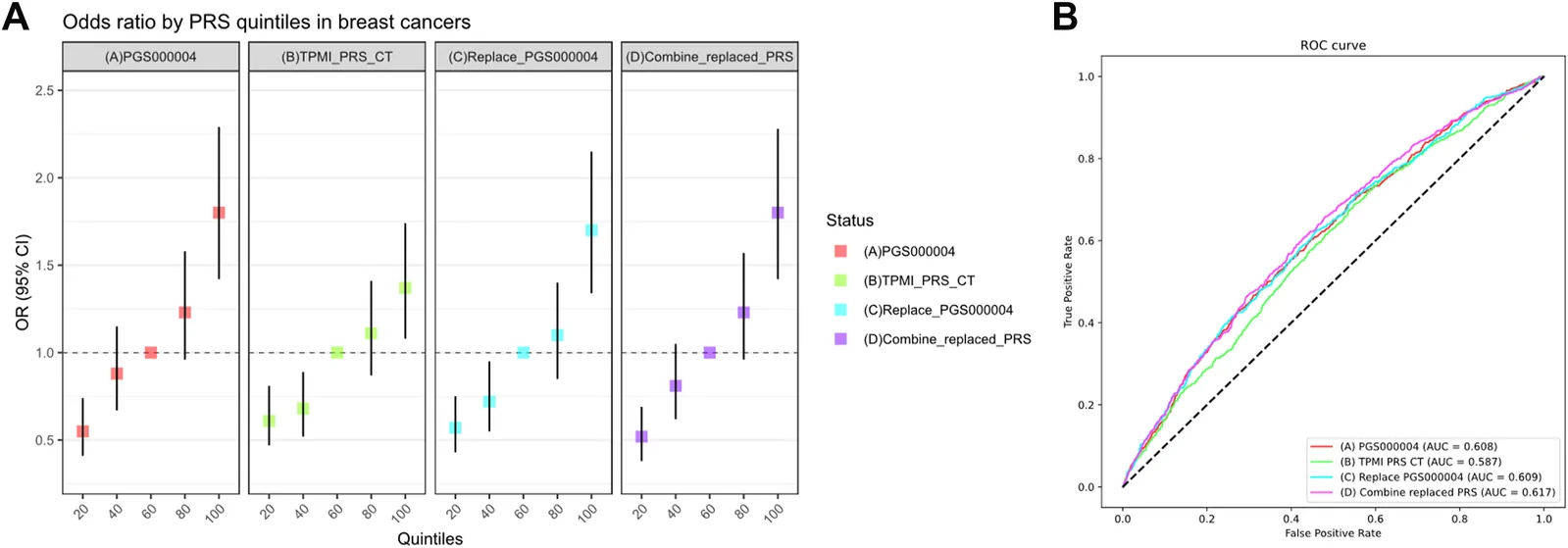
**Performance metrics of polygenic risk scores across different approaches.** (A) Distribution of odds ratios stratified by PRS quintiles. (B) ROC curves show discriminative ability corresponding to the area under the curve (AUC) values. OR, odds ratio; PGS, polygenic score; PRS, polygenic risk score; ROC, receiver operating characteristic; TPMI, Taiwan Precision Medicine Initiative.

### External validation

We demonstrated our approach in an external cohort with 891 individuals. Supplemental Table 3 shows the results of OR_perSD,_ AUC, and OR for the top 5% and 10% individuals in PRS_EUR_, PRS_TPMI,_ PRS_EUR_replace_, and PRS_EUR_replace+TPMI_. The results show that OR_perSD_ is 1.26 (95% CI 1.05-1.50, AUC = 0.559), 1.19 (95% CI 1.00-1.42, AUC = 0.555), 1.37 (95% CI 1.14-1.64, AUC = 0.590), and 1.35 (95% CI 1.13-1.61, AUC = 0.589) for PRS_EUR_, PRS_TPMI,_ PRS_EUR_replace_, and PRS_EUR_replace+TPMI_, respectively. The OR for individuals in the top 5% (2.03, 95% CI 0.84-4.66) and top 10% (1.95, 95% CI 0.99-3.81) is the highest in PRS_EUR_replace+TPMI_. Figure 3 shows the ORs across PRS quantiles and the AUROC for breast cancer risk prediction using different PRS models in the external data set. Supplemental Table 4 provides detailed information on the replaced SNPs and their effect sizes across different LD-*r*^2^ thresholds in the validation data set.

**Figure 3:**
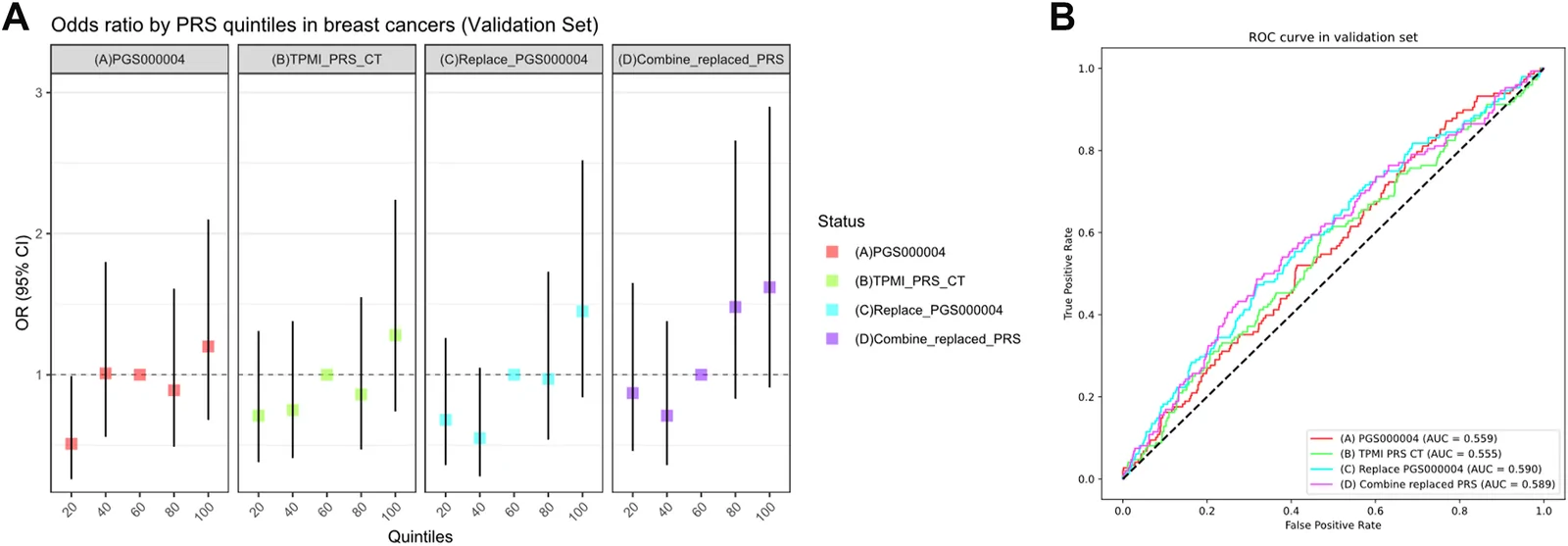
**External validation of polygenic risk scores.** (A) Odds ratios (ORs) across PRS quintiles, demonstrating risk stratification in the validation cohort. (B) ROC curves with corresponding AUROC values in the independent validation data set. AUROC, area under the receiver operating characteristic curve; PGS, polygenic scores; PRS, polygenic risk score; ROC, receiver operating characteristic; TPMI, Taiwan Precision Medicine Initiative.

### Replication in prostate cancer

Our methodology was applied to a cohort of 20,868 individuals, comprising 3478 prostate cancer cases and 17,390 controls (Supplemental Table 2). Our approach demonstrated a similar pattern in predicting the risk of prostate cancer. The combination of PRS derived from European and PRS from our own population with replacement with SNPs within the same LD block yields a highest AUROC of 0.706 and OR_per SD_ of 2.13 (95% CI 1.88-2.42) (Supplemental Table 5). ORs of individuals in each PRS quantile in predicting the risk of developing prostate cancer by different PRSs and the performance of each approach are shown in Supplemental Figure 2.

### Enhancing PRS precision through population-specific genetic variants

Highly similar LD patterns between European and East Asian cohorts were observed (Supplemental Figure 3). Instead, differences in allele frequency appear to be more influential in PRS enhancement when substituting SNPs in the same LD block. We observed substantial minor allele frequency variations between populations, with most substituted SNPs showing higher frequencies in our East Asian cohort than in the European reference cohort (Figure 4A). Specific examples illustrate this trend: rs2290202 was substituted with rs1105291, and rs332529 with rs933688, both demonstrating higher frequencies in the East Asian cohort than in the European cohort. These substituted SNPs exhibited approximately 3-fold higher allele frequencies in our population. The substitution of rs60954078 with rs6913578 revealed the highest effect size among the substituted variants, as visualized in Figure 4B. This finding suggests that effect size estimates play a more crucial role in our SNP substitution methodology. Furthermore, we identified population-specific SNPs for PRS development from our TPMI cohort. Correlation analysis, visualized through R^2^ heatmaps, demonstrated minimal linkage between TPMI-derived SNPs and those from the established PGS000004 catalog (Figure 5). This suggests that population-specific SNPs were identified by using our cohort.

**Figure 4:**
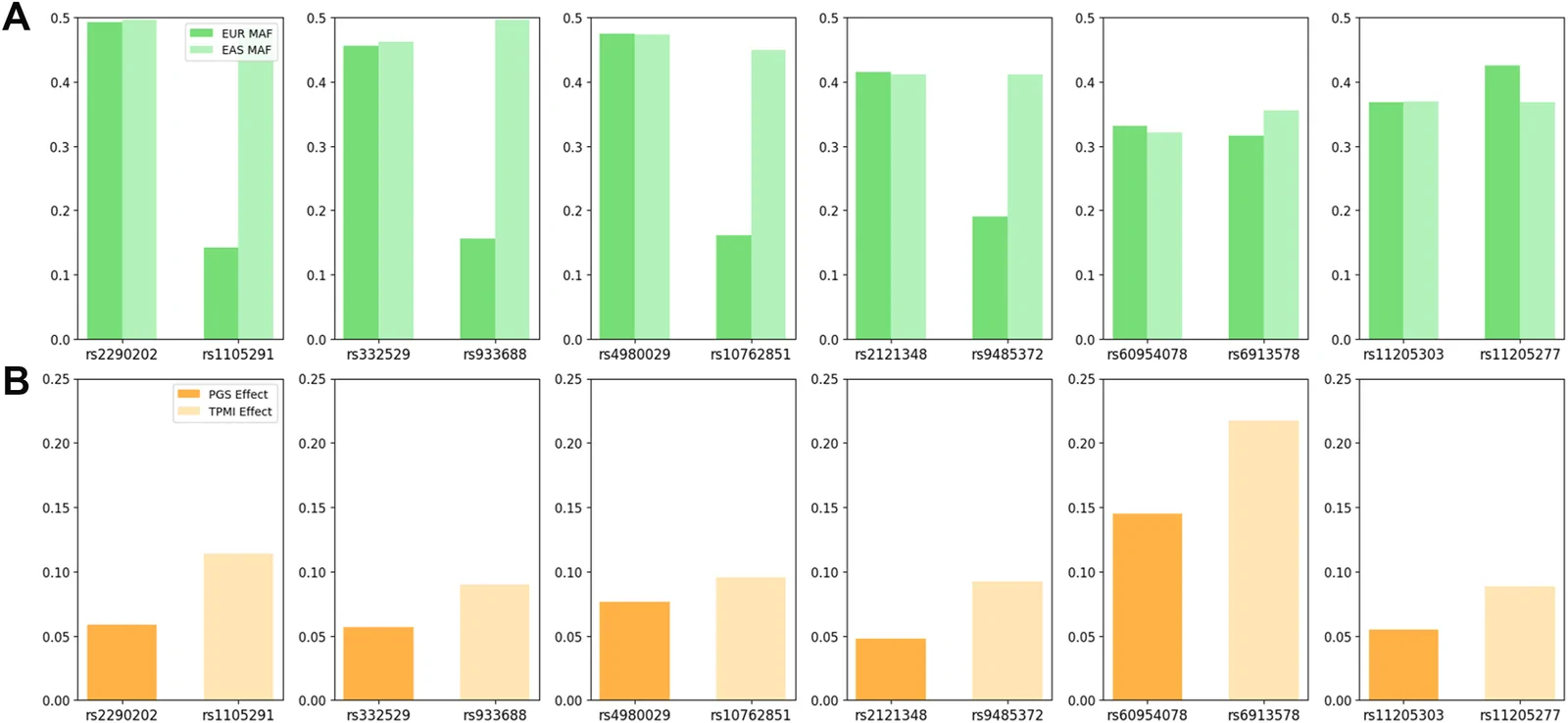
**Population differences in substituted SNP characteristics.** (A) Minor allele frequency (MAF) comparison between European and East Asian cohorts for 6 SNP substitutions. (B) Effect size estimates for each variant across independent studies. MAF, minor allele frequency; PGS, polygenic score; SNP, single-nucleotide polymorphism; TPMI, Taiwan Precision Medicine Initiative.

**Figure 5:**
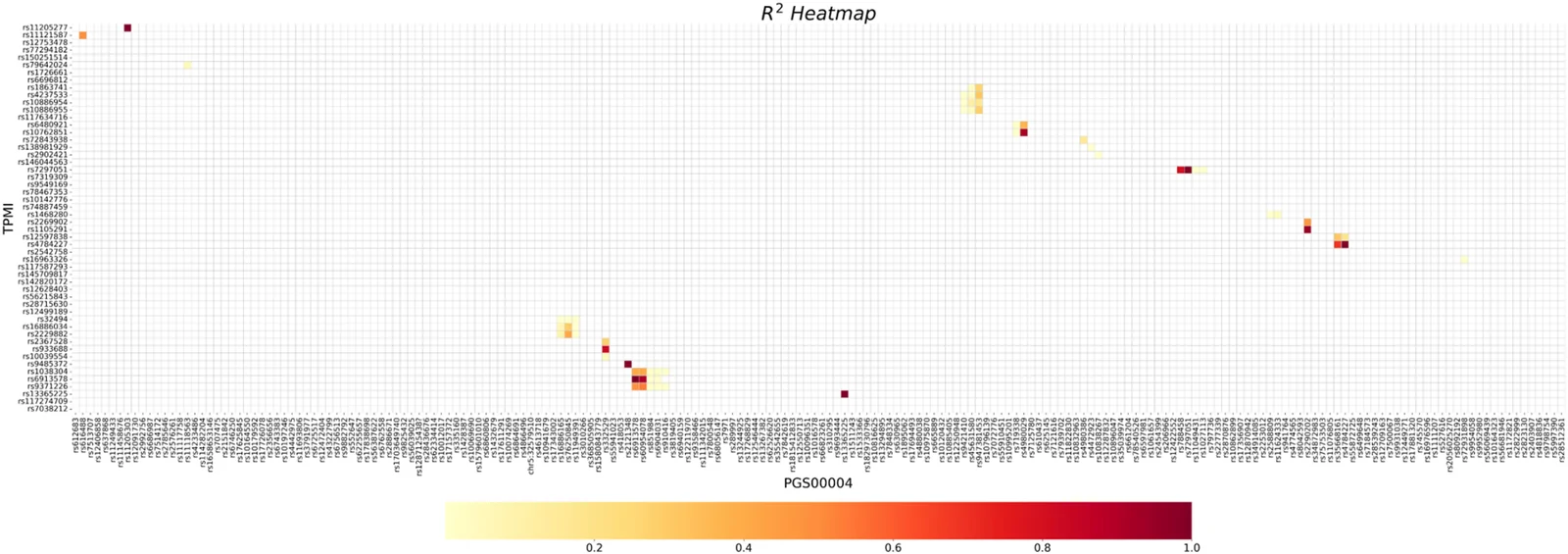
**Correlation heatmap showing linkage disequilibrium (R^2^) between SNPs derived from TPMI and PGS000004 catalog variants.** PGS, polygenic scores; SNP, single-nucleotide polymorphism; TPMI, Taiwan Precision Medicine Initiative.

## Discussion

The predictive performance of PRSs has shown significant advancements in recent years; however, challenges persist in ensuring the generalizability and effectiveness of PRSs across diverse populations. In this study, we present a feasible approach to optimize existing PRSs using a local population to increase predictability, which may accelerate PRS implementation in different ancestry groups. The predominant focus of GWAS on individuals of European descent has resulted in limitations in the predictive accuracy of PRSs when applied to populations with greater genetic divergence from the reference group. Several pipelines have been built that aim to transfer PRS to other populations; however, most of these approaches require a sizable population and still highly rely on the representation of ethnically diverse populations in the data set. A previous study showed that PRSs derived from a combination of European and Asian ancestry GWAS data sets exhibited superior performance compared with PRSs constructed solely using weights obtained from single-ancestry GWAS data.^5^ Our approach provides a potential method to stratify the highest-risk group with improved discrimination, which replaces SNPs in European PRSs with those discovered in the local population within the same LD block. The increase in predictive accuracy, diminishing genetic disparity, may extend the wide applicability and efficacy of PRSs across different ethnicities and populations.

Recent studies have shown substantial genetic overlap in complex traits across ancestries both at variants and genome-wide level, especially between European and East Asian populations,^18^, ^19^, ^20^ suggesting that European-derived PRS can be effectively applied to Asian populations.^3^ However, population-specific genetic architectures, characterized by distinct LD patterns and allele frequency distributions, can impact PRS accuracy across ethnic groups. These differences in genetic architecture are particularly relevant as variants with substantial effect sizes in European populations may exhibit low frequencies in other ancestral groups. This potentially limits their predictive value. Moreover, the development of PRS using non-European cohorts often faces challenges of limited sample sizes and statistical power, leading to potential underdetection of population-specific genetic markers. To address these limitations, we developed a practical approach for optimizing European-derived PRS for application in other populations. Our approach systematically substitutes European PRS variants with population-specific variants identified within the same LD blocks from local data sets. Then, we incorporate additional SNPs uniquely identified in our population cohort. This strategy significantly improves risk stratification, enhancing both the differentiation between cases and controls and the relative risk assessment between top-percentile and intermediate PRS groups. In contrast to prior studies that aim to create a universal cross-population algorithm requiring extensive multiethnic data, our approach effectively uses a smaller local data set to achieve improved generalizability. These results create another opportunity to accelerate the transferability of PRS to diverse populations while large genetic data from underrepresented ethnic groups are still being generated.

In the process of optimizing the PRS, we replaced 6 SNPs from the PRS_EUR_ variants with population-specific variants identified in our cohort, ensuring a linkage disequilibrium threshold of *r*^2^ > 0.8. These substituted SNPs are predominantly linked to breast cancer susceptibility. Analysis of allele frequencies revealed that the original SNPs had frequencies comparable to those in the PRS_EUR_ data set. However, the substituted SNPs demonstrated higher frequencies in our study cohort. Importantly, these substituted SNPs, which were derived from our population, exhibited larger effect sizes for breast cancer risk compared with the SNPs in the PRS_EUR_ data set. While the original PRS_EUR_ has demonstrated consistent risk prediction across more than 10 cohorts through the aggregation of common susceptibility variants, we observed enhanced effect sizes and predictive performance after incorporating our population-specific SNPs. These findings underscore how the current underrepresentation of diverse ethnic populations in genetic studies impacts PRS transferability. Our substitution approach provides an immediate solution for improving PRS performance in non-European populations. However, our results also emphasize the continued importance of expanding genetic data collection in underrepresented populations to better understand the genetic architecture of complex traits across diverse ancestries.

PRS have gained attention for their potential clinical utility in predicting disease risk, including in underrepresented populations where health disparities are prevalent. While PRS offer a promising approach for personalized medicine, concerns have been raised regarding the unequal representation of racial and ethnic minority populations in underlying PRS research.^21^ The lack of genomic information in minorities could lead to the exacerbation of health disparities if PRS are not universally applicable across diverse populations.^22^ Despite efforts that have been made to increase the representation of marginalized racial and ethnic groups in genetic and genomic research, the number of participants is still relatively small compared with individuals of European ancestry. Our approach successfully enhances PRS performance through a straightforward methodology: substituting SNPs within the same LD blocks and incorporating population-specific SNPs identified in our data set. This method offers a more practical alternative to current complex approaches for PRS optimization. By demonstrating the effectiveness of this simplified strategy, our study provides a feasible solution for improving genetic risk prediction in smaller and underrepresented populations. This advancement represents a significant step toward reducing health care disparities and promoting more equitable implementation of precision medicine across diverse populations.

One of the key clinical implications of PRS is their ability to estimate lifetime disease risk, enhancing risk stratification efforts. Previous research has highlighted the impact of integrating PRS into genetic counseling for personalized breast cancer risk assessment.^23^ However, the development of clinical practice guidelines for systematically implementing PRS in breast cancer screening is still evolving, underscoring the need for standardized approaches to optimize their clinical utility.^24^ Our team has also shown the potential of combining PRS with family history, which significantly improves the predictive power for lung cancer risk compared to using family history alone. This study presents a valuable approach, particularly as non-European populations face challenges due to underrepresentation in genetic data. Our method could accelerate the clinical application of PRS in minority populations while large genomic data are still being built.

## Data Availability

The data that support the findings of this study are available from the corresponding author upon request.

## Declaration of Generative AI and AI-Assisted Technologies in the Writing Process

During the preparation of this manuscript, the author(s) used Claude (Anthropic) and Grammarly to improve the grammar and readability of the text. After using these tools, the author(s) reviewed and edited the content as needed and take full responsibility for the content of the published article.

## Conflict of Interest

The authors declare no conflicts of interest.
