# Supplementary material for: Population-specific optimization of polygenic risk scores for breast cancer in East Asians
Source: Genet Med Open. 2026 Aug 3;4:104487. doi: 10.1016/j.gimo.2026.104487 (PMC13627947; doi:10.1016/j.gimo.2026.104487)
Supplement: Supplementary file 1 [file mmc1.docx]

Supplementary Table 1 | Replaced SNPs Table.

| Chromosome | PGS000004 SNPs | TPMI SNPs | GENE symbol  (PGSSNP, TPMISNP) | r2 | MAF-PGS000004 SNP (TPMI) | MAF-TPMI SNP (TPMI) | PGS Effect allele | PGS catalog effect size | TPMI Effect allele | TPMI effect size |
| --- | --- | --- | --- | --- | --- | --- | --- | --- | --- | --- |
| 1 | rs11205303 | rs11205277 | MTMR11, - | 0.98 | 0.3685 | 0.3678 | C | 0.0548 | C | 0.0882 |
| 1 | rs616488 | rs11121587 | PEX14, PEX14 | 0.48 | 0.3309 | 0.2075 | G | -0.0586 | G | -0.1089 |
| 5 | rs332529 | rs933688 | -, - | 0.80 | 0.4561 | 0.4961 | A | -0.0564 | A | -0.0894 |
| 5 | rs76250845 | rs16886034 | -, - | 0.75 | 0.1117 | 0.0992 | T | 0.0865 | C | 0.1593 |
|  |  | rs2229882 | -, MAP3K1 | 0.40 |  | 0.0531 |  | 0.0865 | T | 0.177 |
| 6 | rs2121348 | rs9485372 | TAB2, TAB2 | 0.97 | 0.4152 | 0.4112 | C | -0.0476 | A | -0.092 |
| 6 | rs60954078 | rs6913578 | -, LOC124901435 | 0.90 | 0.3314 | 0.3547 | G | 0.1449 | C | 0.217 |
|  |  | rs9371226 | ESR1 | 0.53 |  | 0.3219 |  |  | T | 0.157 |
|  |  | rs1038304 | CCDC170 | 0.39 |  | 0.4748 |  |  | A | -0.1327 |
| 10 | rs4980029 | rs10762851 | ZMIZ1, ZMIZ1 | 0.90 | 0.4742 | 0.4497 | G | 0.0762 | G | 0.0952 |
|  |  | rs6480921 | ZMIZ1 | 0.34 |  | 0.3275 |  |  | A | -0.0955 |
| 15 | rs2290202 | rs1105291 | PRC1, RCCD1 | 0.93 | 0.492 | 0.484 | T | -0.0589 | T | -0.1141 |
|  |  | rs2269902 | UNC45A & HDDC3 | 0.44 |  | 0.4184 |  |  | C | 0.0866 |
| 16 | rs35668161 | rs4784227 | TOX3, CASC16 | 0.68 | 0.201 | 0.258 | A | 0.1147 | T | 0.2147 |
|  |  | rs4784220 | TOX3 | 0.40 |  | 0.2575 |  |  | C | 0.0937 |
|  |  | rs12597838 | TOX3 | 0.31 |  | 0.2732 |  |  | C | 0.102 |
| 22 | rs5750715 | rs12628403 | -, APOBEC3A | 0.52 | 0.4923 | 0.3841 | A | 0.0407 | C | 0.092 |

Note: 3 SNPs are duplicated in both PGS000004 and TPMI’s SNPs (rs6913578, rs4784227, rs10816625).

Supplementary Table 2 | Basic characteristics of study population in prostate cancer.

|  | All samples after QC step | GWAS  (A,B) | Testing set  (C,E) |
| --- | --- | --- | --- |
| Sample size, n | 20,868 | 34,309 | 2,154 |
| Case, n | 3,478 | 3,119 | 359 |
| Control, n | 17,390 | 31,190 | 1795 |
| Age, mean (SD) | 73.45 (8.36) | 72.84 (8.15) | 71.96 (7.56) |

Supplementary Table 3 | The association of PRSs with breast cancer risk in women in Taiwan using an external validation population.

| PRS | OR_perSD_ (95%CI) | AUROC | OR_Top 5_ (95%CI) | OR_Top 10_ (95%CI) |
| --- | --- | --- | --- | --- |
| (A) PRS_EUR_ | 1.26  (1.05-1.50) | 0.559 | 1.14  (0.44-2.73) | 1.50  (0.77-2.88) |
| (B) PRS_TPMI_ | 1.19  (1.00-1.42) | 0.555 | 1.14  (0.44-2.73) | 1.08  (0.52-2.15) |
| (C-1) PRS_EUR_replace_ | 1.37  (1.14-1.64) | 0.59 | 1.66  (0.70-3.74) | 1.68  (0.87-3.21) |
| (C-2) PRS_EUR_replace10_ | 1.32  (1.11-1.58) | 0.579 | 2.13  (0.88-4.90) | 2.16  (1.09-4.23) |
| (C-3) PRS_EUR_replace17_ | 1.31  (1.10-1.57) | 0.578 | 3.04  (1.25-7.10) | 2.45  (1.19-5.03) |
| (D-1) PRS_EUR_replace+TPMI_ | 1.35  (1.13-1.61) | 0.589 | 2.03  (0.84-4.66) | 1.95  (0.99-3.81) |
| (D-2) PRS_EUR_replace10+TPMI_ | 1.35  (1.14-1.61) | 0.59 | 2.13  (0.88-4.90) | 1.81  (0.90-3.59) |
| (D-3) PRS_EUR_replace17+TPMI_ | 1.36  (1.14-1.62) | 0.59 | 2.13  (0.88-4.90) | 1.69  (0.83-3.39) |

Supplementary Table 4 | The replaced SNP and effect size with different LD-r^2^ in validation set.

|  | (C-1)  PRS_EUR_replace_ | (C-2) PRS_EUR_replace10_ | (C-3) PRS_EUR_replace17_ | (D-1) PRS_EUR_replace+TPMI_ | (D-2) PRS_EUR_replace10+TPMI_ | (D-3) PRS_EUR_replace17+TPMI_ |
| --- | --- | --- | --- | --- | --- | --- |
| LD threshold  Cutoff (≥) | 0.8 | 0.5 | 0.3 | 0.8 | 0.5 | 0.3 |
| Number of SNP | 161 | 162 | 167 | 267 | 265 | 264 |
| Effect size | PGS catalog and TPMI | PGS catalog  and TPMI | PGS catalog and TPMI | PGS catalog  and TPM | PGS catalog  and TPM | PGS catalog  and TPM |
| OR per SD, 95%CI | 1.37  (1.14-1.64) | 1.32  (1.11-1.58) | 1.31  (1.10-1.57) | 1.35  (1.13-1.61) | 1.35  (1.14-1.61) | 1.36  (1.14-1.62) |
| AUROC | 0.590 | 0.579 | 0.578 | 0.589 | 0.590 | 0.590 |
| Top 5% OR | 1.66  (0.70-3.74) | 2.13  (0.88-4.90) | 3.04  (1.25-7.10) | 2.03  (0.84-4.66) | 2.13  (0.88-4.90) | 2.13  (0.88-4.90) |
| Top 10% OR | 1.68  (0.87-3.21) | 2.16  (1.09-4.23) | 2.45  (1.19-5.03) | 1.95  (0.99-3.81) | 1.81  (0.90-3.59) | 1.69  (0.83-3.39) |

Note: Number of SNP listed in this table represent the full set of SNPs used for PRS calculation. Among these, the number of SNPs replaced by cohort-specific variants was as follows: C-1 PRS_EUR_replace_ (6 SNPs), C-2 PRS_EUR_replace10_ (10 SNPs), and C-3 PRS_EUR_replace17_ (17 SNPs).

Supplementary Table 5 | Association of PRSs in a Taiwanese prostate cancer validation cohort.

|  | (A) PRS003415 | (B) TPMI PRS C+T | (C) Replace PRS003415 | (D) Combine the PRS | (E) Combine the replaced PRS |
| --- | --- | --- | --- | --- | --- |
| Method | PRS003415 | Clumping and thresholding R^2^>0.5, p-value<1e-5 | Replace 14 SNPSs with LD threshold r^2^ ≥ 0.8 in (B) | Combine (A) + (B) | Combine (C) + (B) |
| Number of SNP | 197 | 108 | 197 | 300 | 286 |
| Effect size | PGS catalog | TPMI | PGS catalog and TPMI | PGS catalog and TPMI | PGS catalog and TPM |
| OR per SD, 95%CI | 2.11 (1.86-2.39) | 1.88 (1.67-2.12) | 2.08 (1.84-2.35) | 2.13 (1.88-2.41) | 2.13 (1.88-2.42) |
| AUROC | 0.698 | 0.676 | 0.693 | 0.707 | 0.706 |
| Top 5% OR | 6.44 (3.90-10.57) | 3.63 (2.14-6.11) | 5.12 (3.12-8.37) | 4.92 (2.93-8.18) | 4.35 (2.60-7.24) |
| Top 10% OR | 4.50 (2.99-6.79) | 3.79 (2.48-5.80) | 4.58 (3.06-6.84) | 5.21 (3.43-7.93) | 4.53 (2.99-6.87) |

Supplementary Figure 1 | SNP replaced strategy using several LD r2-threshold. The example above demonstrated with Haploview plot of SNPs in LD region on chromosome 1.

Supplementary Figure 2 | Performance of polygenic risk scores. (A) Odds ratios stratified by PRS quintiles, showing risk distribution in a prostate cancer cohort. (B) ROC curves and their corresponding AUROC values in the independent prostate cancer dataset.

Supplementary Figure 3 | Comparative LD patterns of six genomic regions between PGS000004 and TPMI cohorts. Each panel shows paired LD matrices for substituted variants, with PGS000004 patterns displayed in the upper triangle and TPMI cohort patterns in the lower triangle. LD blocks were constructed using all SNPs between the specified variant positions.
